# Adaptive threshold-based alarm strategies for continuous vital signs monitoring

**DOI:** 10.1007/s10877-021-00666-4

**Published:** 2021-02-11

**Authors:** Mathilde C. van Rossum, Lyan B. Vlaskamp, Linda M. Posthuma, Maarten J. Visscher, Martine J. M. Breteler, Hermie J. Hermens, Cor J. Kalkman, Benedikt Preckel

**Affiliations:** 1 Biomedical Signals and Systems, University of Twente, P.O. Box 217, 7500 AE Enschede, The Netherlands; 2 Cardiovascular and Respiratory Physiology, University of Twente, P.O. Box 217, 7500 AE Enschede, The Netherlands; 3 Department of Anesthesiology, University Medical Center Utrecht, Heidelberglaan 100, 3584 CG Utrecht, the Netherlands; 4 Technical Medicine, University of Twente, P.O. Box 217, 7500 AE Enschede, The Netherlands; 5 Department of Anesthesiology, Amsterdam University Medical Center, Location AMC, H1-148, P.O. Box 22660, 1100 DD Amsterdam, The Netherlands

**Keywords:** Vital signs, Clinical alarms, Physiological monitoring, Clinical deterioration, Telemonitoring

## Abstract

Continuous vital signs monitoring in post-surgical ward patients may support early detection of clinical deterioration, but novel alarm approaches are required to ensure timely notification of abnormalities and prevent alarm-fatigue. The current study explored the performance of classical and various adaptive threshold-based alarm strategies to warn for vital sign abnormalities observed during development of an adverse event. A classical threshold-based alarm strategy used for continuous vital signs monitoring in surgical ward patients was evaluated retrospectively. Next, (combinations of) six methods to adapt alarm thresholds to personal or situational factors were simulated in the same dataset. Alarm performance was assessed using the overall alarm rate and sensitivity to detect adverse events. Using a wireless patch-based monitoring system, 3999 h of vital signs data was obtained in 39 patients. The clinically used classical alarm system produced 0.49 alarms/patient/day, and alarms were generated for 11 out of 18 observed adverse events. Each of the tested adaptive strategies either increased sensitivity to detect adverse events or reduced overall alarm rate. Combining specific strategies improved overall performance most and resulted in earlier presentation of alarms in case of adverse events. Strategies that adapt vital sign alarm thresholds to personal or situational factors may improve early detection of adverse events or reduce alarm rates as compared to classical alarm strategies. Accordingly, further investigation of the potential of adaptive alarms for continuous vital signs monitoring in ward patients is warranted.

**Supplementary Information:**

The online version contains supplementary material available at 10.1007/s10877-021-00666-4.

## Introduction

Patients admitted to the hospital for postoperative care are at risk of developing adverse events (AEs), which may lead to serious harm and life threatening situations [1–3]. Early identification and timely treatment of AEs is important to reduce secondary injury and improve patient outcomes [4]. As serious AEs are often preceded by changes in vital signs, routine vital sign measurements are an essential part of early warning systems in the hospital. However, various studies have reported that clinical deterioration in ward patients may be delayed or remain unnoticed due to infrequent or incomplete manual measurements of vital signs [5, 6]. As a result, there is increasing interest in implementing unobtrusive wearable wireless sensors that enable continuous monitoring of vital signs in postoperative patients on the ward and may support early identification of clinical deterioration and AEs [6–10].

Although continuous monitoring has been applied in high care units for many years, its application in the ward is challenged by the lower nurse-to-patient ratio, limited critical care training of nurses, and increased mobilization of patients [11]. In this setting, active alarm systems that support interpretation of signs are crucial for adequate and timely response to potential deterioration [12]. Currently, most mobile monitoring systems adopt traditional and widely used alarm strategies where an alert is sent automatically as soon as measurements of one of the vital signs exceed a pre-set upper or lower threshold. Although this threshold-based alarm system can be life-saving in critical situations [4, 13], it does not consider factors that affect vital signs levels such as physical activity, the circadian rhythm [14] or age [15]. As a result, many false positive alarms are generated in settings where there is continuous (wired) vital signs monitoring, such as intensive care units. Such high false alarm rates are unacceptable for continuous monitoring in a ward setting [9, 16], as alarm overload is a high burden for caregivers and may even cause life-threatening situations from delayed response or even ignored alarms [17, 18]. On the other hand, the use of standard thresholds may result in delayed notification of subtle but relevant vital sign abnormalities. As such, there is a clear clinical need for improved alarm strategies.

Various alternative methods that may improve alarm precision or reduce the number of false alarms in continuous vital signs monitoring have been described, with a clear trend towards intelligent techniques [18–20]. However, the integration of these advanced methods in patient care brings various concerns regarding the accuracy, reliability, efficiency, and interoperability [21–23]. Furthermore, alarms generated by complex or black-box models can be harder to interpret, which may hamper adoption by caregivers. We hypothesize that relatively simple alterations to classical threshold-based strategies may improve identification of AEs in post-surgical ward patients and reduce false alarm rates, which is investigated in the current study.

## Methods

### Data collection

The current observational retrospective study collected data from adult patients that were admitted to the surgical ward for postoperative care after elective major or intermediate surgery in the Amsterdam University Medical Center (Amsterdam, the Netherlands) between December 2018 until March 2019. All patients received standard postoperative care including intermittent vital signs measurements according to local Early Warning Score protocols. In addition, patients were monitored using the wireless Sensium Vitals® system (Sensium Healthcare, Oxford, UK). For this aim, a chest-worn patch sensor with axillary temperature probe was applied to measure the patient’s heart rate (HR), respiratory rate (RR) and axillary temperature (T) every 2 min.

To support continuous monitoring, the original Sensium Vitals® algorithm was used as active alarm system. An alarm was generated in case one of the vital signs measurements exceeded the upper or lower threshold of the predefined normal range (HR: 40–120 beats/min, RR: 8–24 breaths/min, T: ≤ 38 °C respectively) for at least 7 successive measurements. As such, this alarm strategy includes an annunciation delay with interval length of 14 min in case of no missing or invalid measurements. For recurrent abnormalities, a new alarm of the same type was only generated if at least 5 successive measurements (minimal 10 min) had been in the normal range since the preceding alarm. In case of alarms, nurses were asked per protocol to assess the patient. When the nurse judged that an alarm was not caused by technical disturbances or movement, vital signs were measured manually and the Modified Early Warning Score (MEWS) [24] was calculated; further actions were taken according to established local protocols.

Patients were only included for analysis when the total vital signs recording time was at least 24 h and each of the vital signs measurements was available for at least a third of the total recording time. Next to the collection of vital sign measurements and alarms, the presence of observed AEs was assessed retrospectively using the patients clinical record. Adverse events were defined as any postoperative complication, new illness, or deterioration of existing disease described in the patient record. The onset of the AEs was defined as the timing of diagnostic confirmation reported in nursing files, laboratory or radiology results, following the Institute for HealthCare Improvements’ Global Trigger tool [25]. The end of the AE was defined by the moment that AE treatment was no longer reported in the patient record. Only AEs that presented or were treated during the period of continuous monitoring were included in the analysis.

### Simulation of alarm strategies

The collected wireless vital sign measurements of patients and clinically observed alarms were used to retrospectively evaluate the performance of the currently used Sensium Vitals® alarm algorithm for detection of AEs. Next, simulation was used to investigate the performance of alternative alarm strategies in the same dataset. For this aim, the original Sensium Vitals® algorithm was first reproduced retrospectively in MATLAB (version 2019b, The MathWorks Inc., Natick, MA, US) adopting the alarm principles described by the manufacturers and default settings. Subsequently, six alternative alarm strategies were explored by modifying the original alarm algorithm, as specified in Table 1. Two of these strategies were based on previously described methods for abnormality detection (I) or prevention of false alarm rates (III), as explained below. The other strategies were introduced based on physiological assumptions (II, IV, V, VI). For each alternative alarm strategy, three (sets of) parameter settings were subsequently tested to investigate and select optimal standard parameter settings (Table 1). The tested parameter settings were chosen arbitrarily within in a range that was expected suitable, given physiology and default settings of the currently used algorithm.

**Table 1:** Specification and tested parameter settings of alternative alarm strategies

Alternative alarm strategy	Specification	Tested (sets of) parameter settings
I. Threshold individualization	For each individual patient, alarm thresholds are defined using the cumulative density function (CDF), which was reproduced for each vital sign separately using the first 24 h of available data [25]. Accordingly, the standard lower and upper alarm thresholds are replaced by the vital sign level that corresponds to the lower (*CDF*_*low*_) and upper (*CDF*_*high*_) percentiles of the individual CDF. Default alarm thresholds are used for the first 24 h	*- CDF*_*low*_*:* 0.1%; *CDF*_*high*_*:* 99.9% *- CDF*_*low*_*:* 0.5%; *CDF*_*high*_*:* 99.5% *- CDF*_*low*_*:* 1%; *CDF*_*high*_*:* 99%
II. Postoperative elevation of upper thresholds	The standard upper alarm threshold is increased by a fixed percentage (*PO*_*increase*_*,* i.e. postoperative increase factor) for the first four days after surgery	*- PO*_*increase*_*:* 5% for HR/RR; *PO*_*increase*_*:* 1% for T *- PO*_*increase*_*:* 10% for HR/RR; *PO*_*increase*_*:* 2.5% for T *- PO*_*increase*_*:* 25% for HR/RR; *PO*_*increase*_*:* 5% for T
III. Increase annunciation delay interval	The length of the annunciation delay interval (*L*_*interval*_) i.e. minimum number of successive abnormal measurements needed for generation of an alarm (default: 7 measurements, i.e. 14 min interval) is increased	*- L*_*interval*_*:* 12 measurements *- L*_*interval*_*:* 17 measurements *- L*_*interval*_*:* 22 measurements
IV. Daytime elevation of upper HR/RR thresholds	The standard upper HR and RR threshold is increased by a fixed percentage (*DT*_*increase*_ i.e. daytime increase factor) during daytime (8 a.m. to 10 p.m.)	*- DT*_*increase*_: 5% for HR; *DT*_*increase*_*:* 15% for RR *- DT*_*increase*_*:* 10% for HR; *DT*_*increase*_: 25% for RR *- DT*_*increase*_*:* 25% for HR; *DT*_*increase*_: 35% for RR
V. Nighttime reduction of lower HR/RR thresholds	The standard lower HR and RR threshold is decreased by a fixed percentage (*NT*_*increase*_ i.e. nighttime decrease factor) during nighttime (10 p.m. to 8 a.m.)	*- NT*_*decrease*_: 5% for HR; *NT*_*decrease*_*:* 15% for RR *- NT*_*decrease*_: 10% for HR; *NT*_*decrease*_*:* 25% for RR *- NT*_*decrease*_*:* 25% for HR; *NT*_*decrease*_*:* 35% for RR
VI. Slope-based alarms	An alarm is generated only in case the slope of the linear regression line calculated over a past time interval (*T*_*slope*_) exceeds a preset threshold: HR slope: ± 15 bpm over *T*_*slope*_ RR slope: ± 10 brpm over *T*_*slope*_ T slope: ± 1 °C over *T*_*slope*_	*- T*_*slope*_*:* 4 h *- T*_*slope*_*:* 8 h *- T*_*slope*_*:* 12 h

*HR* heart rate, *RR* respiratory rate, *T* axillary temperature, *bpm* beats per minute, *brpm* breaths per minute

The first alternative alarm strategy (I) implemented individual thresholds to correct for differences in normal vital signs ranges between patients. For this aim, the first available 24 h of the recording was used to create individual distributions of the vital signs for each patient and identify corresponding upper and lower alarm thresholds for the remaining monitoring period, similar to the approach described by Poole et al. [26].

The second alarm strategy (II) aimed to prevent false alarms, by increasing upper threshold levels in the first four postoperative days where levels for HR, RR and T are typically higher due to the surgical stress response [27, 28].

The third strategy (III) focused on optimization of the annunciation delay, supported by the beneficial results reported in other studies [20, 29, 30]. Accordingly, an increase in the interval length of alarms was simulated, such that vital signs should exceed a threshold for a longer successive period to cause an alarm. With this adaptation, it was aimed to reduce the number of false alarms related to short lasting abnormalities caused by normal variations or movement artifacts.

The fourth (IV) alarm strategy was designed to compensate for increased physical activity level, which leads to increased HR and RR levels as compared to resting state. As patients are most active during daytime, the upper HR and RR threshold was increased for daytime (8 a.m. to 10 p.m.) to prevent false alarms.

Likewise, the fifth alarm strategy (V) corrected for low HR and RR levels that are often observed during sleep [31] by decreasing the corresponding lower threshold during nighttime (10 p.m. to 8 a.m.).

The sixth alarm strategy (VI) assessed vital signs solely based on time trends, as patterns of change are crucial in the detection of clinical deterioration [32]. Accordingly, this alarm strategy generated alarms in case the upward or downward slope calculated over a predefined time window exceeded a certain threshold, without taking the absolute vital sign value into account. Trends were assessed for time windows of multiple hours, as the wireless monitoring system is currently indicated for detection of clinical deterioration and not as surveillance system for acute situations.

### Evaluation of alarm strategies

The alarms that were respectively generated in clinical practice or during simulation were defined as true positives (TP) or false positives (FP) to evaluate the performance to detect AEs. Alarms that occurred in the 24 h before diagnostic confirmation and during the treatment period of the AE were classified as TP in case the vital sign abnormality could be physiologically explained by development or presence of the AE. To enable consequent alarm classification, a list of assumed relations between AEs and vital sign abnormalities was composed using clinical guidelines and literature. In case subsequent AEs with overlapping windows of presentation were observed, alarms that could be related to both events were not double counted but allocated only to the event that developed latest in time. As continuous monitoring is aimed to be used as an early warning tool, TP alarms that were generated in the 24 h before diagnostic confirmation of the AE were also investigated as a separate category (TP_early_).

The performance of the original alarm strategy and each of the optimized alternative strategies was evaluated using two sensitivity rates (S_total_, S_early_), the total alarm rate, and the false discovery rate. S_total_ and S_early_ were defined as the number of AEs for which TP alarms or TP_early_ were observed respectively, and represent the sensitivity for detection or early detection of AEs. The total alarm rate was calculated as the sum of all alarms divided by the total recording time of all patients, resulting in an average number of alarms/day/patient. The false discovery rate was calculated as the percentage of alarms classified as FP. In addition to these four metrics, we introduced a performance score (P-score) to evaluate the relative improvement in overall performance for each of the alternative alarm strategies as compared to the original alarm strategy, based on the trade-off between early AE detection and total alarm rate. For this aim, sub scores were assigned to the level of increase or decrease in S_early_ and total alarm rate, as specified in Table 2. The P-score was calculated as the sum of the two sub scores assigned to S_early_ and total alarm rate respectively. Accordingly, a positive P-score indicates improvement in overall performance as opposed to the original alarm strategy the while a negative P-score indicates impairment.

**Table 2:** Scores used to calculate the performance score (P-score)

Score	Sensitivity for early detection (S_early_ (%))	Total alarm rate (TAR (alarms/patient/day))
−3	S_early_ ≤ S_early:ref_–10	TAR > TAR_ref_ + 0.5
−2	S_early_−10 < S_early_ ≤ S_early:ref_−5	TAR_ref_ + 0.25 < TAR ≤ TAR_ref_ + 0.5
−1	S_early:ref_−5 < S_early_ ≤ S_early:ref_	TAR_ref_ < TAR ≤ TAR_ref_ + 0.25
0	S_early_ = S_early:ref_	TAR = TAR_ref_
1	S_early:ref_ < S_early_ ≤ S_early:ref_ + 5	TAR_ref_–0.25 < TAR ≤ TAR_ref_
2	S_early:ref_ + 5 < S_early_ ≤ S_early:ref_ + 10	TAR_ref_–0.5 < TAR ≤ TAR_ref_ – 0.25
3	S_early_ > S_early:ref_ + 10	TAR ≤ TAR_ref_–0.5

S_early_: sensitivity for early detection of adverse events, S_early:ref_: sensitivity of original alarm strategy for early detection of adverse events (reference). TAR: total alarm rate, TAR_ref_: total alarm rate of original alarm strategy (reference). The performance score (P-score) is calculated as the sum of the two scores that correspond to the S_early_ and total alarm rate, respectively

For each alternative alarm strategy, the parameter set with highest P-score was selected as most optimal and used as standard setting applied to each patient record for further analysis and evaluation. In case of an equal P-score, the setting with lowest false discovery rate or the setting with smallest modification (lowest correction factor) as compared to the original alarm algorithm was selected subsequently. In addition to evaluation of individual alarm strategies, we explored whether combining multiple strategies improved alarm performance. For this aim, all possible combinations of strategies I to V were implemented cumulatively. The trend-based strategy (VI) was not included in these combinations due to its incompatibility with strategies that adapt thresholds for absolute vital sign values. Last, stepwise backward elimination was performed. Accordingly, the strategies that affected the P-score most were removed step-by-step from the combination, starting from the full combination of strategies (I–V). This process was repeated until all combination sizes were tested.

## Results

### Patients

Data was collected for a total of 60 patients, of which 21 patients were excluded due to limited availability of wireless vital signs recordings. Table 3 reports the characteristics of the 39 remaining patients that were included for analysis.

**Table 3:** Population characteristics (N=39)

Baseline characteristics	N (%)
Male gender	21 (54)
Age (years)	62 (51–72)
Physical status	
ASA I	1 (3)
ASA II	28 (72)
ASA III	9 (23)
Unknown	1 (3)
Type of surgery	
Upper gastrointestinal	8 (21)
Lower gastrointestinal	12 (31)
Other abdominal	8 (21)
Other	1 (3)
Comorbidities	
Gastrointestinal	23 (59)
Cardiac	9 (23)
Pulmonary	9 (23)
Diabetes	5 (13)
Other	6 (15)
None	1 (3)
Length of hospital stay (days)	9 (6–12)
Adverse events observed	
Anastomotic leak	3 (8)
Pneumonia	3 (8)
Chyle leak	2 (5)
Wound infection	2 (5)
Atelectasis	1 (3)
Atrial fibrillation	1 (3)
Hydropneumothorax	1 (3)
Hypocalcemia after thyroid surgery	1 (3)
Ileus	1 (3)
Pericarditis	1 (3)
Pulmonary embolism	1 (3)

Values represent the number (%) of patients or the median (interquartile range) value. *ASA* american society of anesthesiologists physical status classification

A total of 20 included patients (51%) developed one or more AEs during postoperative ward stay. In 14 patients, AEs presented during the continuous monitoring period, resulting in a total inclusion of 18 AEs (Clavien Dindo class I: N = 6, II: N = 8, III: N = 4). The type of included AEs is reported in Table 3.

### Current alarm strategy

In total, 3999 h of vital signs data were available for the 39 included patients with a median duration of 94 (range: 28–279) h per patient. The population distribution of the vital signs is shown in Supplementary file 1 (Fig. 3). The original Sensium Vitals® algorithm generated a total of 83 alarms in 20 out of 39 patients, which translates to an average total alarm rate of 0.49 (median: 0.18, IQR: 0.0–0.73) alarms per patient per day.

**Fig. 1:**
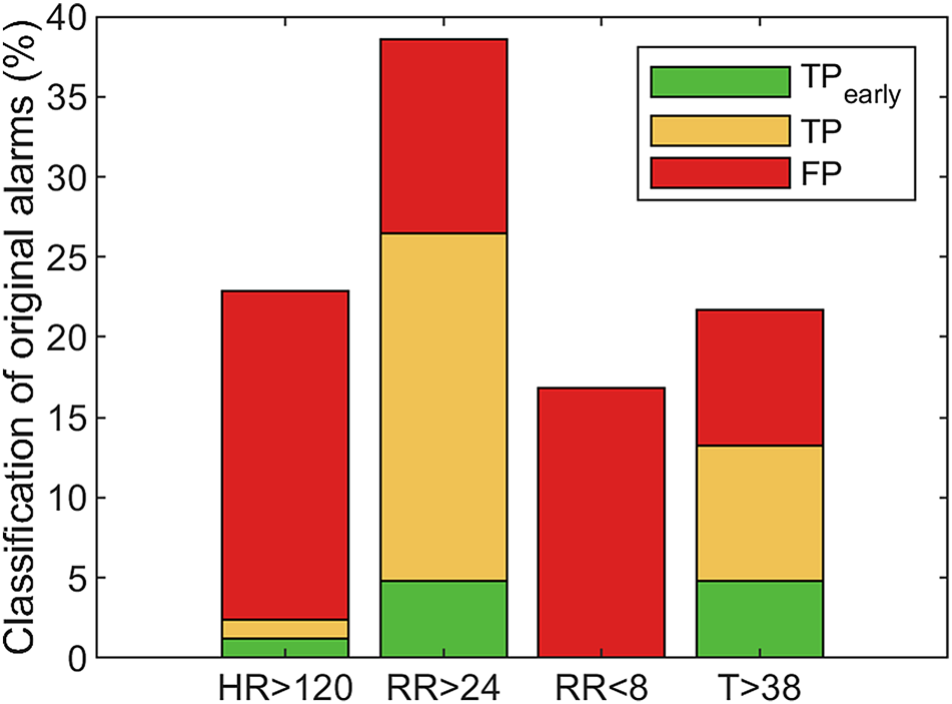
Classification of the alarms (N = 83) generated by the clinical alarm system in included patient population (N = 39).* HR* heart rate,* RR* respiratory rate,* T* axillary temperature,* TP* True positive alarm. TP_early_: true positive alarm presenting before presentation of the adverse event.* FP* False positive alarm. No alarms were observed for low HR values (HR < 40)

Figure 1 reports the type and classification of original alarms observed, indicating clear differences in the total amount of HR, RR, and T alarm types and the corresponding ratio of TP and FP alarms. Most alarms (63%) presented during daytime (8 a.m–10 p.m). Furthermore, the false discovery rate during daytime (52%) was lower as compared to nighttime (68%), which indicates that daytime alarms were more often classified as TP alarms. Often, alarms were not spread throughout the admission period but presented clustered on a specific day. Days with ≥ 3 alarms were found in seven patients.

**Fig. 2:**
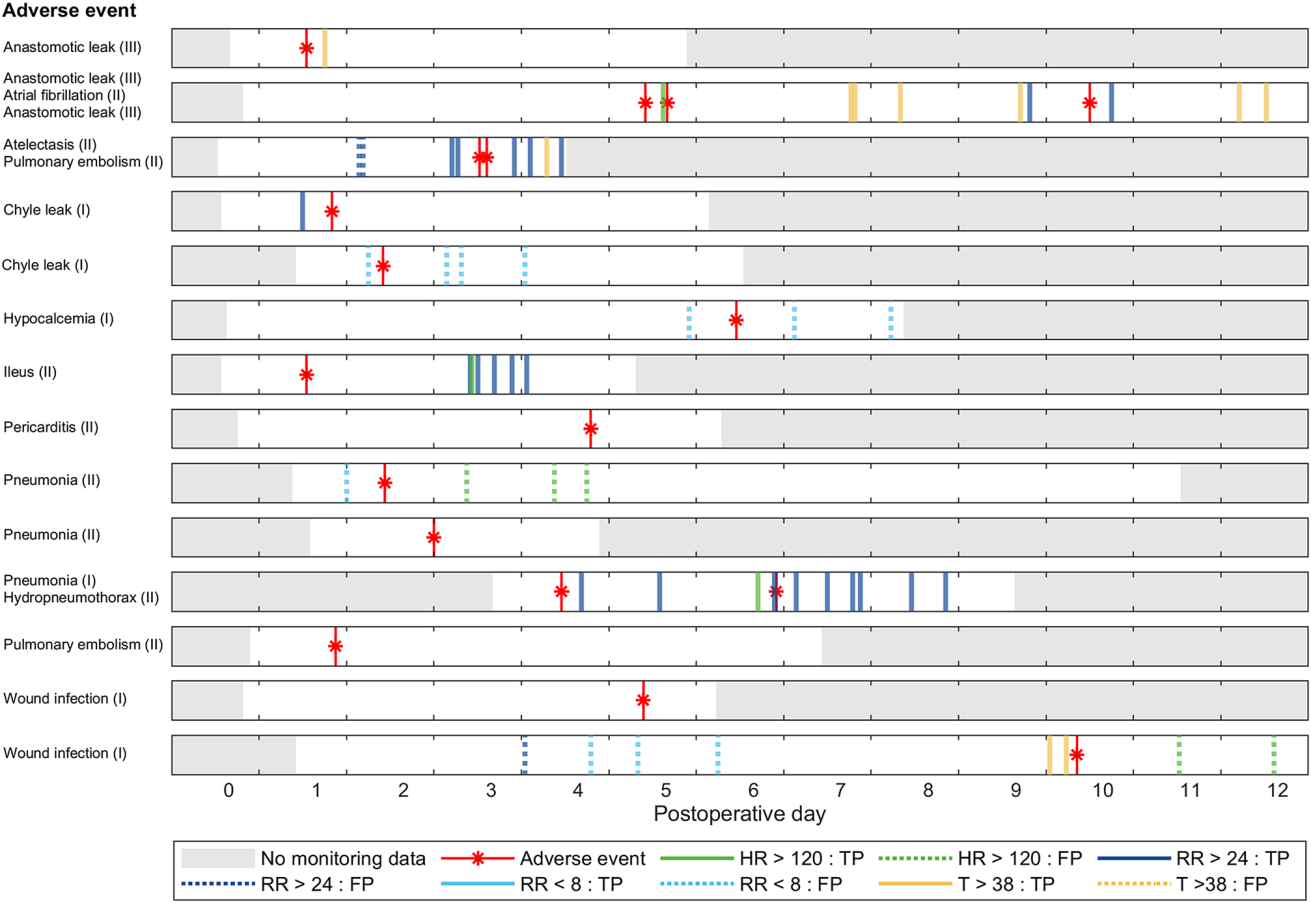
Timing and type of alarms observed in patients (N = 14) with adverse events during continuous monitoring on the ward.* HR* heart rate,* RR* respiratory rate,* T* axillary temperature,* TP* True positive alarm.* FP* False positive alarm. I–III: Clavien Dindo classification. The monitoring period is shown up to 12 days after surgery, since no adverse events or alarms were observed in later periods

Figure 2 visualizes the presentation of different alarm types for patients with observed AEs. In total, 42% of the alarms generated by the original algorithm were classified as TP, where one or more TP alarms were observed in 11 out of 18 AEs (S_total_: 61%). In seven of these AEs, TP alarms were caused by one type of vital sign (HR, RR or T) only. However, the type of alarm was not necessarily the same for the few AE types that were observed in multiple patients (see Fig. 2). TP alarms were exclusively triggered by high vital signs levels and never for low levels. Although most TP alarms were generated during the period of AE treatment, alarms were generated before diagnostic confirmation in seven AEs (S_early_: 39%). TP alarms were observed for all four AEs with Clavien Dindo score of III, but only in half of the AEs with Clavien Dindo score of I (3 out of 6) or II (4 out of 8). In 15 out of 25 (60%) of the patients without events, no alarms were generated at all.

### Alternative alarm strategies

The reproduced original algorithm that was used as starting point to simulate alternative alarm strategies regenerated 96% of the original alarms and created two additional alarms that were not created by the original algorithm. Table 4 summarizes the performance of the six simulated alternative alarm strategies, using the parameter settings that provided optimal results out of three tested options. The performance of all settings can be found in Supplementary file 2 (Table 6). As compared to the original alarm strategy, adapted alarm strategy I and VI implemented with the optimal settings improved overall detection and early identification of AEs (S_early_ and S_total_) but also led to multifold increase in total alarm rate. In contrast, adapted alarm strategy II, III and IV decreased the total alarm rate at the cost of S_early_ and S_total_. Strategy V led to a lower daily alarm rate without affecting (early) detection of AEs, constituting the only strategy with a positive P-score. This reduction in total alarm rate was only related to the modification in the lower threshold of RR as no alarms were generated for low HR.

**Table 4:** Performance of original and alternative alarm strategies

Alarm strategy	Optimal parameter set	S_early_ (% of AEs preceded by TP_early_ alarms)	S_total_ (% of AEs with TP alarms)	Total alarm rate (alarms/patient/day)	False detection rate (% of alarms classified as false positive)	P-score
Original	N.A.	39	61	0.49	59	N.A.
I. Threshold individualization	*CDF*_*low*_: 1%; *CDF*_*high*_: 99%	56	78	1.81	83	0
II. Postoperative elevation of upper thresholds	*PO*_*increase*_: 5% for HR/RR; *PO*_*increase*_: 1% for T	33	56	0.42	45	−1
III. Increase annunciation delay interval	*L*_*interval*_: 12 measurements	33	50	0.25	50	0
IV. Daytime elevation of upper HR/RR thresholds	*DT*_*increase*_: 5% for HR; *DT*_*increase*_: 15% for RR	33	50	0.35	66	−1
V. Nighttime reduction of lower HR/RR thresholds	*NT*_*decrease*_: 10% for HR; *NT*_*decrease*_: 25% for RR	39	61	0.45	55	1
VI. Slope-based alarms	*T*_*slope*_: 4 h	50	78	3.47	94	0

For definition of alarm strategies (I-VI) and corresponding parameters see Table 1. S_total_: sensitivity for detection of adverse events, S_early_: sensitivity for early detection of adverse events, P-score: performance score (for specification see Table 2), *AE* adverse event (N=18), *TP* true positive alarm, *TP*_early_ true positive alarm presenting before presentation of the adverse event, *NA* not applicable

The performance of all tested combinations is found in Supplementary file 2 (Table 7), and Table 5 reports the results of the backward selection process. Various combinations of strategies improved overall performance (P-score ≥ 1), which was always the result of a larger increase in S_early_ relative to the growth in total alarm rate. Although S_early_ increased most in the well-performing alarm strategies, this was often accompanied by higher levels of S_total_ as well. The combination of strategy II, III and IV performed best and increased S_early_ to 61% and S_total_ to 72%, but also caused a small increase in total alarm rate to 0.59 alarms/patient/day. Remarkably, all combinations with high P-score (P-score = 2) included strategy II, whilst this strategy impaired performance when implemented solely. Strategy I contributed least to improving alarm performance, as this strategy was included least frequently in combinations with improved performance and dropped out first in the backward elimination process.

**Table 5:** Performance of combined alternative alarm strategies

I	II	III	IV	V	S_early_ (% of AEs preceded by TP_early_ alarms) (%)	S_total_ (% of AEs with TP alarms) (%)	Total alarm rate (alarms/patient/day)	False detection rate (% of alarms classified as false positive) (%)	P-score
×	×	×	×	×	56	78	2.33	91	0
	×	×	×	×	56	67	0.55	75	2
	×	×		×	61	72	0.59	70	2
	×	×			61	72	0.62	71	2

I–V: number of alternative alarm strategy (for definition see Table 1) implemented using optimal parameter settings (as mentioned in Table 4). The crosses indicate that the considering alternative alarm strategy was included in the combination. S_total_: sensitivity for detection of adverse events, S_early_: sensitivity for early detection of adverse events, P-score: performance score (for specification see Table 2)

## Discussion

### Main findings

This study evaluated the performance of classical and adaptive threshold-based alarm strategies for continuous vital signs monitoring in ward patients. We aimed to explore easy-to-implement and transparent methods to support identification of clinical deterioration related to postoperative AEs. Our results show that the currently used classical threshold-based alarm strategy detected abnormalities in vital signs before or after onset of treatment in most of the observed AEs in ward patients. Each of the six adapted threshold-based alarm strategies that we simulated retrospectively in the same population showed potential to either increase the sensitivity for detection of AEs or to reduce the total alarm rate as compared to the currently used alarm strategy. However, the individual alarm strategies caused minimal improvement or even impairment of overall alarm performance. Combining specific alternative alarm strategies improved overall performance most, where sensitivity rates increased while raising only few extra alarms. In particular, the number of AEs where alarms were observed in the 24 h prior to onset of treatment was increased, which suggests that implementation of multiple approaches to adaptive alarm thresholds may improve early detection of clinical deterioration in ward patients.

### Evaluating alarm strategies

Alarms are seen as an essential element of continuous physiological monitoring, as these support timely identification of abnormalities and create awareness of potentially relevant deterioration. Yet, the alarm burden is also considered as one of the major concerns for successful implementation of continuous monitoring in a ward setting [9]. Therefore, critical evaluation of optimal alarm strategies for this setting is desired. Although there is general consensus about the need for adequate alarm systems, no clear definition of acceptable alarm rates and situations that require alarms exist. By definition, alarm systems are most effective in case the alerts promote actions that directly or indirectly contribute to patient outcome. Furthermore, it is known that the response towards alarms is best in case alarms convey specific events [16, 33]. Most studies that investigated detection methods for ward patients used cardiac arrest, ICU transfer, or death as marker for deterioration [19, 34], where the need to call for rapid action is obvious. However, we believe that alarm strategies for ward patients should also focus on less severe events that are more common in this setting, and to the early phase of serious AEs where sequelae could still be minimized. Accordingly, the current study evaluated alarm strategies by their ability to detect any type of postoperative adverse event requiring treatment, focusing on actionable situations.

Despite the small study cohort, we were able to study a relatively high rate [3] and variety of AEs. By using a retrospective study set-up and investigation of alarms that presented in the 24-h window prior to AE treatment, we explored whether alarms could serve as an early warning tool. However, one should be aware that most AEs develop gradually which hampers sharp limitation of the corresponding onset and duration, challenged even more by variations in clinical response times and delays in reporting. Furthermore, it should be kept in mind that vital sign measurements do not detect diseases but only signs of deterioration related to (progression of) disease. Therefore, vital sign abnormalities that develop in a later phase of AEs may also be of clinical importance. For this reason, early alarms as well as alarms that presented after onset of AE treatment were included in the evaluation of overall TP rate. Still, as the causality of vital sign abnormalities and true timing of AEs remains uncertain, careful interpretation of sensitivity rates is required.

### Relation to previous studies

Even though various methods to improve alarm strategies for continuous vital signs monitoring have been described [20], most monitoring systems still work with classical thresholds-based alarms and high rates of alarms remain problematic as today [17, 35]. The average alarm rate observed in the current study was approximately 0.5 alarms per patient per day, which is markedly low as compared to previously reported rates of physiological monitoring systems used in the ICU (38–350 alarms/patient/day) and ward (96 alarm/patient/day) [36, 37]. Although this lower alarm rate is partially explained by the fact that currently used monitoring system does not assess oxygen saturation, blood pressure and electrocardiogram, this also indicates that current alarm strategy has relative good performance in terms of minimizing alarm burden. Still, more than half of the observed alarms was classified as false positive and no abnormalities were detected for a part of the AEs, supporting the search for improvement of alarm strategies.

Various studies described that manual or automated personalization of alarm thresholds improves alarm strategies for vital signs monitoring [26, 38]. Besides, its has been suggested that use of trend information contributes to outcome prediction [32, 39]. In the current study, the personalized and trend-based strategies (I and VI) were indeed able to improve sensitivity rates but also resulted in relatively high alarm rates. These findings indicate that the isolated assessment of relative or absolute changes in vital sign levels has limited specificity for AE detection, and question whether normal ranges should solely be based on previous postoperative measurements of the individual patient and without considering current vital sign levels. As such, further investigation of alternative methods that define and adapt to normal patterns representative for an unaffected physiological state are warranted.

As expected, adapting the annunciation delay interval (strategy III) reduced alarm rates, which was in line with previous studies [20, 29, 30]. Likewise, the methods correcting for the postoperative phase (strategy II) or day/night differences (strategy IV and V) lowered the number of alarms. Still, most of these strategies also reduced sensitivity rates when applied individually, and resulted in minimal improvement or even impairment of overall alarm performance. Combining strategy II–V was more effective and led to highest performance scores observed in current study, supporting the expectation that integration of different methods is beneficial [20].

However, remarkably, combining strategy II–V improved sensitivity rather than alarm rates, which is the opposite effect as observed for individual implementation of strategy II, III, IV or V. These reversed results indicate that the overall benefits of the modifications strongly depend on the overall algorithm design and settings applied, which is possible related to the general limitations of static single-parameter alarms. This is underlined by studies reporting that classical methods for detection of deterioration are outperformed by more advanced methods for personalization of alarm thresholds [38] or identification of abnormal trends or patterns in vital signs [19, 32]. Furthermore, the integration of vital signs and context data can improve prediction of severe outcome events, which has led to the development of various patient assessment tools such as the MEWS [24], electronic Cardiac Arrest Risk Triage (eCART) score [40], Rothman score [41], and prediction methods based on machine learning [42–44]. However, most of these methods used more complex models or require additional data sources, and their clinical benefits still have to be demonstrated for applications of continuous wireless patient monitoring. Nevertheless, their underlying principles may guide further improvement of adaptive systems that trigger clinical response.

### Limitations

To simulate modification to the original algorithm, the current clinically used alarm strategy was reconstructed based on descriptions of the original source code. Although the alarms of the reproduced original algorithm were almost identical to those observed in clinical practice, some inaccuracy may have been induced. Furthermore, even though accuracy of the currently used wireless monitoring system has been described as acceptable and reliable for HR and RR monitoring in ward patients [45], the continuous measurements could have been affected by missing data or inaccuracies. As such, the performance of the clinically used alarm strategy and adapted alarm methods may not translate to other systems and requires external verification.

To evaluate modified alarm strategies and optimize parameter settings, we introduced a performance score assessing the degree of improvement in sensitivity rate and total alarm rate as compared to the original algorithm. However, the optimal trade-off between sensitivity and alarm load in a ward setting is not yet established [20]. Besides, the specificity of alarm strategies is also relevant but could not be judged, as the number of true and false negative cases could not be verified retrospectively. Last, the opportunities to improve alarm performance were limited due to the relatively small population size, low clinical alarm rate, and by restricting the number of alterations and range of parameter settings that was tested. In addition, the methods used in the adaptive strategies were based on previously described principles or physiological assumptions, and the computational design or settings were not further trained or adapted for individual patients. Furthermore, the trend-based strategy was only tested in isolation, while combined or stepped assessment of trends and absolute vital sign values may be of interest as well. Therefore, larger prospective studies are desired to further optimize and integrate the alternative threshold-based alarm strategies and validate current results. Moreover, it is recommended to evaluate the effects of the adaptive alarm strategies also in other settings where higher false alarm rates are currently observed. Last, it is desired to verify the performance of the adaptive alarm strategies in relation to alternative methods for prediction of patient deterioration such as the MEWS score, and to assess their overall potential clinical benefits.

## Conclusions

In conclusion, a classical threshold-based alarm strategy is able to identify abnormalities in continuously measured vital signs for the majority of AEs observed in surgical ward patients without causing excessive alarm rates. Implementation of transparent methods that adapt thresholds to personal or situational factors may increase event detection rates or lower alarm rates as compared to the classical strategy, yet with no or minimal overall improvement of alarm performance. Combining multiple adaptive threshold-based strategies seems more successful in improving alarm performance and may contribute to increased or earlier identification of clinical deterioration.

## Supplementary Information

Below is the link to the electronic supplementary material.

Supplementary file1 (PDF 53 KB)

Supplementary file2 (PDF 111 KB)

## References

[1] Zegers M, de Bruijne MC, de Keizer B, Merten H, Groenewegen PP, van der Wal G (2011). The incidence, root-causes, and outcomes of adverse events in surgical units: implication for potential prevention strategies. Patient Saf Surg.

[2] Anderson O, Davis R, Hanna GB, Vincent CA (2013). Surgical adverse events: a systematic review. Am J Surg.

[3] The International Surgical Outcomes Study group (2016). Global patient outcomes after elective surgery: prospective cohort study in 27 low-, middle-and high-income countries. Br J Anaesth.

[4] DeVita MA, Smith GB, Adam SK, Adams-Pizarro I, Buist M, Bellomo R (2010). “Identifying the hospitalised patient in crisis”—A consensus conference on the afferent limb of Rapid Response Systems. Resuscitation.

[5] Ludikhuize J, Smorenburg SM, de Rooij SE, de Jonge E (2012). Identification of deteriorating patients on general wards; measurement of vital parameters and potential effectiveness of the Modified Early Warning Score. J Crit Care.

[6] Taenzer AH, Spence BC (2018). The afferent limb of rapid response systems: continuous monitoring on general care units. Crit Care Clin.

[7] Boer C, Touw HR, Loer SA (2018). Postanesthesia care by remote monitoring of vital signs in surgical wards. Curr Opin Anesthesiol.

[8] Breteler MJM, KleinJan E, Numan L, Ruurda JP, Van Hillegersberg R, Leenen LPH (2020). Are current wireless monitoring systems capable of detecting adverse events in high-risk surgical patients? A descriptive study. Injury.

[9] Downey CL, Chapman S, Randell R, Brown JM, Jayne DG (2018). The impact of continuous versus intermittent vital signs monitoring in hospitals: A systematic review and narrative synthesis. Int J Nurs Stud.

[10] Khanna AK, Hoppe P, Saugel B (2019). Automated continuous noninvasive ward monitoring: future directions and challenges. Crit Care.

[11] Posthuma LM, Visscher MJ, Hollmann MW, Preckel B (2019). Monitoring of high- and intermediate-risk surgical patients. Anesth Analg.

[12] Vincent J-L, Einav S, Pearse R, Jaber S, Kranke P, Overdyk FJ (2018). Improving detection of patient deterioration in the general hospital ward environment. Eur J Anaesthesiol.

[13] Kellett J, Sebat F (2017). Make vital signs great again – A call for action. Eur J Intern Med.

[14] Krauchi K, Wirz-Justice A (1994). Circadian rhythm of heat production, heart rate, and skin and core temperature under unmasking conditions in men. Am J Physiol Integr Comp Physiol.

[15] Chester JG, Rudolph JL (2011). Vital Signs in Older Patients: Age-Related Changes. J Am Med Dir Assoc.

[16] McGrath SP, Taenzer AH, Karon N, Blike G (2016). Surveillance monitoring management for general care units: strategy, design, and implementation. Jt Comm J Qual Patient Saf.

[17] Cvach M (2012). Monitor alarm fatigue: an integrative review. Biomed Instrum Technol.

[18] Hravnak M, Pellathy T, Chen L, Dubrawski A, Wertz A, Clermont G (2018). A call to alarms: current state and future directions in the battle against alarm fatigue. J Electrocardiol.

[19] Petit C, Bezemer R, Atallah L (2018). A review of recent advances in data analytics for post-operative patient deterioration detection. J Clin Monit Comput.

[20] Winters BD, Cvach MM, Bonafide CP, Hu X, Konkani A, O’Connor MF (2018). Technological distractions (part 2): a summary of approaches to manage clinical alarms with intent to reduce alarm fatigue. Crit Care Med.

[21] da Costa CA, Pasluosta CF, Eskofier B, da Silva DB, da Rosa Righi R (2018). Internet of health things: toward intelligent vital signs monitoring in hospital wards. Artif Intell Med.

[22] Baig MM, GholamHosseini H, Moqeem AA, Mirza F, Lindén M (2017). A systematic review of wearable patient monitoring systems – current challenges and opportunities for clinical adoption. J Med Syst.

[23] Neill DB (2013). Using artificial intelligence to improve hospital inpatient care. IEEE Intell Syst.

[24] Subbe CP, Kruger M, Rutherford P, Gemmel L (2001). Validation of a modified Early Warning Score in medical admissions. QJM.

[25] Griffin FA, Resar RK (2009). IHI global trigger tool for measuring adverse events (Second Edition).

[26] Poole S, Shah N (2018). Addressing vital sign alarm fatigue using personalized alarm thresholds. Pacific Symp Biocomput.

[27] Hollis RH, Graham LA, Lazenby JP, Brown DM, Taylor BB, Heslin MJ (2016). A role for the early warning score in early identification of critical postoperative complications. Ann Surg.

[28] Finnerty CC, Mabvuure NT, Ali A, Kozar RA, Herndon DN (2013). The surgically induced stress response. J Parenter Enter Nutr.

[29] Welch J, Kanter B, Skora B, McCombie S, Henry I, McCombie D (2016). Multi-parameter vital sign database to assist in alarm optimization for general care units. J Clin Monit Comput.

[30] Paine CW, Goel VV, Ely E, Stave CD, Stemler S, Zander M (2016). Systematic review of physiologic monitor alarm characteristics and pragmatic interventions to reduce alarm frequency. J Hosp Med.

[31] Posthuma LM, Visscher MJ, Lirk PB, van Dijkum EJMN, Hollmann MW, Preckel B (2019). Insights into postoperative respiration by using continuous wireless monitoring of respiratory rate on the postoperative ward: a cohort study. J Clin Monit Comput.

[32] Brekke IJ, Puntervoll LH, Pedersen PB, Kellett J, Brabrand M (2019). The value of vital sign trends in predicting and monitoring clinical deterioration: a systematic review. PLoS One.

[33] Chopra V, McMahon LF (2014). Redesigning hospital alarms for patient safety: alarmed and potentially dangerous. Jama.

[34] Gao H, McDonnell A, Harrison DA, Moore T, Adam S, Daly K (2007). Systematic review and evaluation of physiological track and trigger warning systems for identifying at-risk patients on the ward. Intensive Care Med.

[35] Drews FA 2008. Patient monitors in critical care: Lessons for improvement. In: Henriksen K, Battles JB, Keyes MA, Grady ML, editors. Adv patient Saf new Dir Altern approaches (vol 3 Perform tools) Agency Healthc Res Qual (US), Rockville, Maryl. 21249940

[36] Imhoff M, Kuhls S (2006). Alarm algorithms in critical care monitoring. Anesth Analg.

[37] Gross B, Dahl D, Nielsen L (2011). Physiologic monitoring alarm load on medical/surgical floors of a community hospital. Biomed Instrum Technol.

[38] Tarassenko L, Clifton DA, Pinsky MR, Hravnak MT, Woods JR, Watkinson PJ (2011). Centile-based early warning scores derived from statistical distributions of vital signs. Resuscitation.

[39] Churpek MM, Adhikari R, Edelson DP (2016). The value of vital sign trends for detecting clinical deterioration on the wards. Resuscitation.

[40] Bartkowiak B, Snyder AM, Benjamin A, Schneider A, Twu NM, Churpek MM (2019). Validating the electronic cardiac arrest risk triage (eCART) score for risk stratification of surgical inpatients in the postoperative setting: retrospective cohort study. Ann Surg.

[41] Rothman MJ, Rothman SI, Beals J (2013). Development and validation of a continuous measure of patient condition using the Electronic Medical Record. J Biomed Inform.

[42] Churpek MM, Edelson DP (2016). Moving beyond single parameter early warning scores for rapid response system activation. Crit Care Med.

[43] Clifton L, Clifton DA, Pimentel MAF, Watkinson PJ, Tarassenko L (2014). Predictive monitoring of mobile patients by combining clinical observations with data from wearable sensors. IEEE J Biomed Heal Informatics.

[44] Churpek MM, Yuen TC, Winslow C, Meltzer DO, Kattan MW, Edelson DP (2016). Multicenter comparison of machine learning methods and conventional regression for predicting clinical deterioration on the wards. Crit Care Med.

[45] Breteler MJM, KleinJan EJ, Dohmen DAJ, Leenen LPH, van Hillegersberg R, Ruurda JP (2020). Vital signs monitoring with wearable sensors in high-risk surgical patients: a clinical validation study. Anesthesiology.

