# Supplementary material for: Adaptive threshold-based alarm strategies for continuous vital signs monitoring
Source: J Clin Monit Comput. 2021 Feb 11;36(2):407–17. doi: 10.1007/s10877-021-00666-4 (PMC9123069; doi:10.1007/s10877-021-00666-4)
Supplement: Supplementary file 1 — Supplementary file1 (PDF 53 KB) [file 10877_2021_666_MOESM1_ESM.pdf]

Supplementary file 1

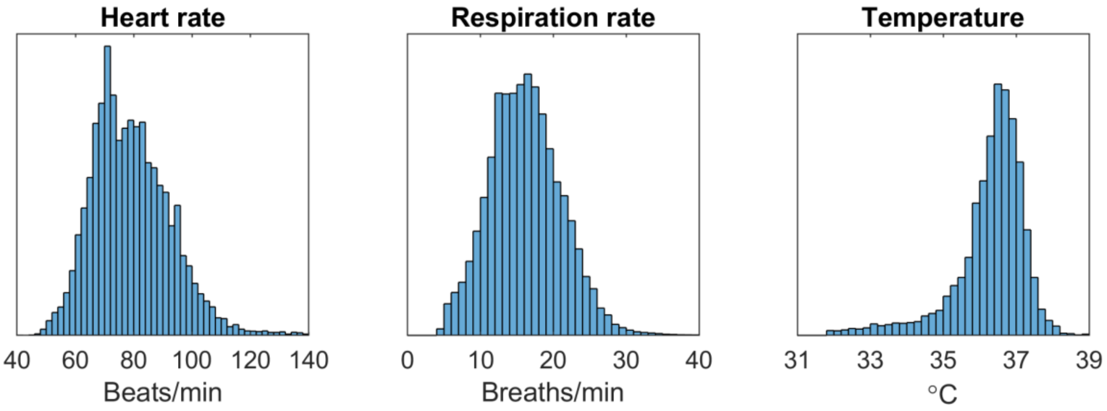

Fig. 3 Distribution of continuous heart rate, respiratory rate, and temperature measurements obtained in the included surgical ward patients (N = 39)
