# Supplementary material for: Adaptive threshold-based alarm strategies for continuous vital signs monitoring
Source: J Clin Monit Comput. 2021 Feb 11;36(2):407–17. doi: 10.1007/s10877-021-00666-4 (PMC9123069; doi:10.1007/s10877-021-00666-4)
Supplement: Supplementary file 2 — Supplementary file2 (PDF 111 KB) [file 10877_2021_666_MOESM2_ESM.pdf]

Supplementary file 2

Table 6 Performance of original and alternative alarm strategies

Strategy	Parameter setting	S_{early} (% of AEs preceded by TP_{early} alarms)	S_{total} (% of AEs with TP alarms)	Total alarm rate (alarms/ patient/ day)	False detection rate (% of alarms classified as false positive)	P-score
Original	Default	39	61	0.49	59	N.A.
I. Threshold individualization	CDF_{low}: 0.1%<br>CDF_{high}: 99.9%	44	61	0.92	84	-1
	CDF_{low}: 0.5%<br>CDF_{high}: 99.5%	50	67	1.44	84	0
	CDF_{low}: 1%<br>CDF_{high}: 99%	56	78	1.81	83	0*
II. Postoperative elevation of upper thresholds	$PO_{increase}$: 5% for HR/RR<br>$PO_{increase}$: 1% for T	33	56	0.42	45	-1*
	$PO_{increase}$: 10% for HR/RR<br>$PO_{increase}$: 2.5% for T	28	56	0.40	40	-2
	$PO_{increase}$: 25% for HR/RR<br>$PO_{increase}$: 5% for T	22	33	0.35	41	-2
III. Increase annunciation delay interval	$L_{interval}$: 12 samples	33	50	0.25	50	0*
	$L_{interval}$: 17 samples	28	44	0.16	50	-1
	$L_{interval}$: 22 samples	28	44	0.14	52	-1
IV. Daytime elevation of upper HR/RR thresholds	$DT_{increase}$: 5% for HR<br>$DT_{increase}$: 15% for RR	33	50	0.35	66	-1*
	$DT_{increase}$: 10% for HR<br>$DT_{increase}$: 25% for RR	28	50	0.32	67	-2
	$DT_{increase}$: 25% for HR<br>$DT_{increase}$: 35% for RR	22	44	0.29	63	-2
V. Nighttime reduction of lower HR/RR thresholds	$NT_{increase}$: 5% for HR<br>$NT_{increase}$: 15% for RR	39	61	0.46	55	1
	$NT_{increase}$: 10% for HR<br>$NT_{increase}$: 25% for RR	39	61	0.45	55	1*
	$NT_{increase}$: 25% for HR<br>$NT_{increase}$: 35% for RR	39	61	0.45	55	1
VI. Slope-based alarms	T_{slope}: 4 h	50	78	3.5	94	0*
	T_{slope}: 8 h	6	17	0.7	97	-4
	T_{slope}: 12 h	6	17	0.3	88	-2

For definition of alarm strategies (I–VI) and corresponding parameters see Table 1 of the manuscript. S_{total}: sensitivity for detection of adverse events, S_{early}: sensitivity for early detection of adverse events, P-score: performance score (for specification see Table 2 of manuscript), AE: adverse event (N=18), TP: True positive alarm, TP_{early}: true positive alarm presenting before presentation of the adverse event, N.A: Not applicable. *selected as setting with best performance

Table 7 Performance of combined alternative alarm strategies

I	II	III	IV	V	S_{early} (% of AEs preceded by TP_{early} alarms)	S_{total} (% of AEs with TP alarms)	Total alarm rate (alarms/ patient/ day)	False detection rate (% of alarms classified as false positive)	P-score
x	x	x	x	x	56%	78%	2.33	91%	0
x	x	x	x		56%	78%	2.31	91%	0
x	x	x		x	56%	78%	2.12	92%	0
x	x		x	x	67%	83%	3.03	91%	0
x		x	x	x	44%	56%	0.85	80%	1
	x	x	x	x	56%	67%	0.55	75%	2
x	x	x			56%	78%	2.10	91%	0
x	x			x	67%	83%	2.83	92%	0
x			x	x	61%	72%	1.53	83%	0
		x	x	x	28%	44%	0.18	50%	-1
	x	x	x		56%	67%	0.58	76%	2
x		x		x	56%	72%	1.03	81%	0
	x		x	x	61%	72%	0.65	74%	2
x		x	x		44%	56%	0.83	80%	1
x	x		x		67%	83%	2.96	91%	1
	x	x		x	61%	72%	0.59	70%	2
x	x				67%	83%	2.75	91%	0
x		x			56%	72%	1.01	81%	0
x			x		61%	72%	1.45	83%	0
x				x	61%	78%	1.89	84%	0
	x	x			61%	72%	0.62	71%	2
	x		x		61%	72%	0.69	76%	2
	x			x	67%	78%	0.76	67%	1
		x	x		28%	44%	0.20	56%	-1
		x		x	33%	50%	0.23	45%	0
			x	x	33%	50%	0.30	60%	-1

I–V: number of alternative alarm strategy (for definition see Table 1 of manuscript) implemented using optimal parameter settings (as mentioned in Table of manuscript). The crosses indicate that the considering alternative alarm strategy was included in the combination. S_{total}: sensitivity for detection of adverse events, S_{total}: sensitivity for detection of adverse events, S_{early}: sensitivity for early detection of adverse events, P-score: performance score (for specification see Table 2 of manuscript), N.A: Not applicable
